# High risk of breast cancer in women with biallelic pathogenic variants in *CHEK2*

**DOI:** 10.1007/s10549-020-05543-3

**Published:** 2020-01-28

**Authors:** Irene Rainville, Shanell Hatcher, Eric Rosenthal, Katie Larson, Ryan Bernhisel, Stephanie Meek, Heidi Gorringe, Erin Mundt, Susan Manley

**Affiliations:** Myriad Genetics, Inc., 320 Wakara Way, Salt Lake City, UT 84108 USA

**Keywords:** *CHEK2*, Breast cancer, Germline, Biallelic

## Abstract

**Purpose:**

Compared to breast cancer risk genes such as *BRCA2*, *ATM*, *PALB2,* and *NBN*, no defined phenotype is currently associated with biallelic pathogenic variants (PVs) in *CHEK2*. This study compared the prevalence of breast and other cancers in women with monoallelic and biallelic *CHEK2* PVs.

**Methods:**

*CHEK2* PV carriers were identified through commercial hereditary cancer panel testing (09/2013–07/2019). We compared cancer histories of 6473 monoallelic carriers to 31 biallelic carriers. Breast cancer risks were estimated using multivariate logistic regression and are reported as odds ratios (OR) with 95% confidence intervals (CI).

**Results:**

Breast cancer frequency was higher among biallelic *CHEK2* PV carriers (80.6%, 25/31) than monoallelic carriers (41.2%, 2668/6473; *p* < 0.0001). Biallelic carriers were more likely to be diagnosed at or before age 50 (61.3%, 19/31) and to have a second breast cancer diagnosis (22.6%, 7/31) compared to monoallelic carriers (23.9%, 1548/6473; *p* < 0.0001 and 8.1%, 523/6473; *p* = 0.0107, respectively). Proportionally more biallelic carriers also had any cancer diagnosis and > 1 primary diagnosis. Compared to women with no PVs, biallelic PV carriers had a higher risk of developing ductal invasive breast cancer (OR 8.69, 95% CI 3.69–20.47) and ductal carcinoma in situ (OR 4.98, 95% CI 2.00–12.35) than monoallelic carriers (OR 2.02, 95% CI 1.90–2.15 and OR 1.82, 95% CI 1.66–2.00, respectively).

**Conclusions:**

These data suggest that biallelic *CHEK2* PV carriers have a higher risk for breast cancer, are more likely to be diagnosed younger, and to have multiple primary breast cancers compared to monoallelic carriers. Biallelic carriers also appear to have a higher risk of cancer overall. Therefore, more aggressive management may be appropriate for women with biallelic PVs in *CHEK2* compared with current recommendations for monoallelic carriers.

## Introduction

*CHEK2* is considered a moderate risk breast cancer gene, with estimates of the relative risk for women carrying a single pathogenic variant (PV) ranging from 2.0 to 4.8 for a first breast cancer. The risk for a second primary breast cancer following an initial diagnosis is estimated to be increased 2.8- to 3.5-fold over individuals with breast cancer without pathogenic variants (PVs) in breast cancer risk genes [1–4]. An increased risk of colorectal cancer has also been reported for *CHEK2* PV carriers, however the evidence for this association is not well established [5–7]. More recent evidence supports a possible association with other cancers such as testicular germ cell tumors [8], renal cell cancer [9], and lethal prostate cancer [10]. Despite known and possible cancer associations for *CHEK2* PV carriers, phenotypic differences between monoallelic and biallelic carriers are not yet understood. Biallelic carriers of PVs in other, dominant breast cancer susceptibility genes such as *BRCA2*, *ATM*, *PALB2*, and *NBN* are known to have more severe cancer phenotypes than monoallelic carriers [11–13]. Similar patterns for *CHEK2* PV carriers have yet to be adequately established.

A single *CHEK2* founder mutation, c.1100del, is present in 1.1% of individuals of Northern and Eastern European ancestry [14]. Assuming Hardy–Weinberg equilibrium, roughly 3/10000 of Northern and Eastern Europeans will be homozygous for c.1100del, with the possibility of additional biallelic individuals if other *CHEK2* PVs are included. Homozygous *CHEK2* c.1100del has, in fact, been reported in 14 female breast cancer cases in the Dutch population. In one study of 8 female homozygous carriers, the risk of breast cancer was estimated to be twice the risk in heterozygous carriers [15]. Because the number of homozygous carriers in this study was small, the calculated confidence intervals (CI) for breast cancer risk were wide, making the interpretation of these findings challenging. Similar results were observed in another study of six homozygous cases [16].

Based on the evidence of increased breast cancer risk in women with monoallelic *CHEK2* PVs, current NCCN guidelines recommend annual mammography and consideration of breast MRI beginning at age 40 [12]. It is important to establish if women who are homozygous for *CHEK2* c.1100del or are biallelic carriers of any *CHEK2* PV have higher breast cancer risks than monoallelic carriers, since this could impact management recommendations. In order to better assess the cancer risks in rare female carriers of biallelic *CHEK2* PVs, we identified *CHEK2* PV carriers in a large series of patients undergoing hereditary cancer panel testing. We then compared the prevalence of breast cancer and other cancers in women with biallelic and monoallelic *CHEK2* PVs.

## Methods

### Patients and hereditary cancer testing

Monoallelic and biallelic female carriers of *CHEK2* PVs were identified through clinical pan-hereditary cancer panel testing at a Clinical Laboratory Improvement Amendments and College of American Pathologists-approved commercial laboratory (Myriad Genetic Laboratories, Inc., Salt Lake City, UT) between September 2013 and July 2019. The panel test included at least 25 genes (*APC, ATM, BARD1, BMPR1A, BRCA1, BRCA2, BRIP1, CDH1, CDK4, CDKN2A, CHEK2, EPCAM, MLH1, MSH2, MSH6, MUTYH, NBN, PALB2, PMS2, PTEN, RAD51C, RAD51D, SMAD4, STK11*, and *TP53*) or up to 35 genes (with the step-wise addition of *GREM1, POLD1,* and *POLE,* followed by *HOXB13,* and then *AXIN2, GALNT12, MSH3, NTHL1, RNF43,* and *RPS20*). All patients provided informed consent for clinical testing. De-identified clinical information was obtained from provider-completed test request forms (TRFs). Data was included only for women residing in states with no legal prohibitions on the use of de-identified data for research purposes.

### Variant analysis, phase determination, and statistical methods

Variants were classified according to the American College of Medical Genetics and Genomics Guidelines supplemented with additional statistical algorithms [17, 18]. The *CHEK2* founder mutations c.470C > T (p.Ile157Thr) and c.1283C > T (p.Ser428Phe) were not included in this analysis, as these were classified as variants of uncertain significance by the laboratory during the study time period and this analysis was restricted to PVs [19]. *CHEK2* PV carriers who had a PV in another gene were also excluded. Phase in biallelic carriers was determined by allelic haplotypes or by confirming carrier status in relatives. Biallelic carriers with first-degree relatives confirmed to have only one PV were considered confirmed to carry PVs *in trans.* Fisher’s exact tests for difference in proportions were used to determine differences between monoallelic and biallelic *CHEK2* carriers. *p*-values < 0.05 were considered significant. Adjustments for multiple comparisons were not performed as this analysis was hypothesis-driven.

A previously-published multivariable logistic regression model was used to estimate breast cancer risks [4]. A separate analysis was performed for breast cancer according to subtype with *CHEK2* PV carrier status (monoallelic and biallelic) as a dependent variable. Independent variables included personal (binary flags) and family (numeric counts weighted by degree of relative) cancer histories of breast (lobular invasive, ductal invasive, ductal carcinoma in situ [DCIS], and male), ovarian, colorectal, melanoma, gastric, pancreatic, prostate, endometrial, and colon (polyps) as well as age at testing, sex and ancestry. 95% confidence intervals (CIs) were calculated using Wald statistics.

## Results

### Cohort description

In this study, we identified 6473 monoallelic and 42 biallelic carriers of *CHEK2* PVs. Of those 42 biallelic CHEK2 PV carriers, phase was confirmed to be *in trans* for 31 cases, 16 of which were homozygous for *CHEK2* c.1100del. In this cohort, the majority of *CHEK2* PV carriers were of White/Non-Hispanic origin, both in monoallelic and biallelic carriers (69.8% and 71.0%, respectively; Table 1). Among biallelic PV carriers, there appeared to be a possible over-representation of patients with Hispanic/Latino ancestry compared to among monoallelic carriers (12.9% and 5.9%, respectively). On average, biallelic carriers of *CHEK2* PVs were diagnosed with breast and other cancers at younger ages than monoallelic carriers.

**Table 1:** Cohort demographics

Characteristic	Variable	Monoallelic (*N* = 6473)	Biallelic^a^ (*N* = 31)
Age at testing (years)	Range	16, ≥ 90	27, 74
	Median (IQR)	48 (38, 58)	51 (40, 63)
	≤ 50	56.3%	48.4%
Age at cancer diagnosis (any cancer)^b^	Range	1, ≥ 90	24, 66
	Median (IQR)	47 (40, 56)	44 (37.5, 49.5)
Age at breast cancer diagnosis^c^	Range	16, ≥ 90	30, 66
	Median (IQR)	48 (42, 57)	44 (38, 49)
Ancestry	White/Non-Hispanic	4521 (69.8%)	22 (71.0%)
	Hispanic/Latino	384 (5.9%)	4 (12.9%)
	Black/African	87 (1.3%)	0
	Ashkenazi Jewish	68 (1.1%)	0
	Middle Eastern	34 (0.5%)	1 (3.2%)
	Asian	33 (0.5%)	0
	Other^d^	65 (1.0%)	0
	Multiple	352 (5.4%)	2 (6.5%)
	Not provided	929 (14.4%)	2 (6.5%)
Personal cancer history^e^	Breast	2668 (41.2%)	25 (80.6%)
	Ovarian	230 (3.6%)	2 (6.5%)
	Endometrial	117 (1.8%)	0
	Colorectal	97 (1.5%)	0
	Melanoma	98 (1.5%)	0
	Pancreatic	10 (0.2%)	1 (3.2%)
	Gastric/stomach	2 (< 0.1%)	0
	Colorectal polyps	201 (3.1%)	0
	Non-colorectal polyps	1 (< 0.1%)	0
	Other	441 (6.8%)	3 (9.7%)
	No cancer history	3234 (50.0%)	3 (9.7%)

^a^Confirmed *in trans*

^b^Only includes patients with a cancer diagnosis (28 biallelic PV carriers, 3239 monoallelic PV carriers)

^c^Only includes patients with a breast cancer diagnosis (25 biallelic PV carriers, 2668 monoallelic PV carriers)

^d^Other includes Native American, Pacific Islander, and all other ancestries

^e^Rows are not exclusive; patients could have a personal history of multiple cancers

### Cancer prevalence in monoallelic and biallelic *CHEK2* PV carriers

Only 9.7% (3/31) of biallelic *CHEK2* PV carriers were reported with no history of cancer, compared to 50.0% (3234/6473) of monoallelic *CHEK2* PV carriers (Table 1). Breast cancer was the most prevalent cancer reported in both monoallelic and biallelic carriers of *CHEK2* PVs, with ovarian cancer being the second most prevalent single cancer in both. The frequency of at least one primary breast cancer at any age was significantly (*p* < 0.0001) higher in biallelic carriers (80.6%, 25/31) than in monoallelic carriers (41.2%, 2668/6473; Table 2). Biallelic CHEK2 PV carriers were significantly more likely to be diagnosed with breast cancer at or before age 50 (61.3%, 19/31), as well as to have a second breast cancer diagnosis (22.6%, 7/31), as compared to monoallelic carriers (23.9%, 1548/6473; *p* < 0.0001 and 8.1%, 523/6473; *p* = 0.0107, respectively). The increased prevalence of breast cancer, breast cancer at age 50 or younger, and second primary breast cancer was also observed when the comparisons were restricted to monoallelic and homozygous carriers of *CHEK2* c.1100del (Table 2). No excess of any specific non-breast cancer was clearly identified in the biallelic *CHEK2* PV carriers overall, though pancreatic cancer did appear to be more common among biallelic and *CHEK2* c.1100del homozygous carriers (3.2% and 6.2%, respectively) as compared to monoallelic and *CHEK2* c.1100del monoallelic carriers (0.2%; *p* = 0.0512 and 0.1%; *p* = 0.0277, respectively).

**Table 2:** Breast cancer frequency in *CHEK2* PV carriers

Variable	Monoallelic	Biallelic	*p*-value	c.1100del Monoallelic	c.1100del Homozygous	*p*-value
Breast cancer (any age)	2668 (41.2%)	25 (80.6%)	< 0.0001	1403 (41.1%)	13 (81.2%)	0.0015
Breast cancer (≤ 50 years)	1548 (23.9%)	19 (61.3%)	< 0.0001	799 (23.4%)	9 (56.2%)	0.0048
Breast cancer (any age) + 2nd primary breast cancer	523 (8.1%)	7 (22.6%)	0.0107	280 (8.2%)	5 (31.2%)	0.0078
Breast cancer (any age) + any non-breast cancer	324 (5.0%)	3 (9.7%)	0.2027	188 (5.5%)	3 (18.8%)	0.0558

**Fig. 1:**
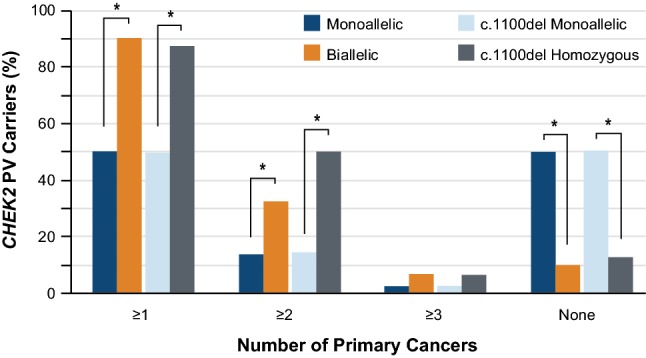
Distribution of women with one or two *in trans* PVs in *CHEK2* according to number of primary cancers of any type. *p < 0.05

The prevalence of multiple cancers among monoallelic and biallelic *CHEK2* PV carriers was also compared. Biallelic *CHEK2* PV carriers were significantly more likely to be affected with at least one primary cancer as compared to monoallelic PV carriers (90.3% vs. 50.0%, *p* < 0.0001; Fig. 1). Similarly, biallelic *CHEK2* PV carriers were significantly more likely to have at least two primary cancers when compared to monoallelic PV carriers (32.3% vs. 13.5%, *p* = 0.0061). These differences were also significant for carriers of homozygous *CHEK2* c.1100del PVs as compared to monoallelic *CHEK2* c.1100del carriers. The converse comparison of unaffected monoallelic and biallelic *CHEK2* PV carriers revealed that biallelic carriers were significantly less likely to be unaffected with cancer as compared to monoallelic carriers (*p* < 0.0001). Similarly, 50.4% of *CHEK2* c.1100del monoallelic carriers were unaffected while only 12.5% of *CHEK2* c.1100del homozygous carriers were unaffected by any cancer (*p* = 0.0022).

### Age-related cancer penetrance in monoallelic and biallelic *CHEK2* PV carriers

**Fig. 2:**
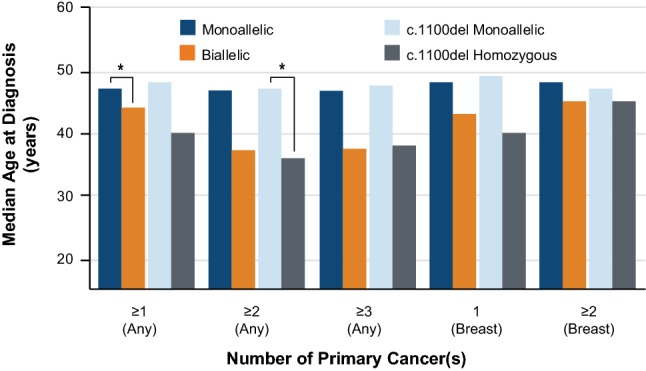
Median age at cancer diagnosis in women with one or two *in trans* CHEK2 PVs*.* *p < 0.05

The median age of first cancer diagnosis was compared in monoallelic and biallelic *CHEK2* PV carriers for any cancer, and for breast cancer specifically. A trend toward younger median age at cancer diagnosis was observed in biallelic *CHEK2* PV carriers as compared to monoallelic carriers for all evaluated categories (≥ 1, ≥ 2, or ≥ 3 cancer diagnoses of any kind; 1 or ≥ 2 breast cancer diagnosis), though this trend was generally not statistically significant (Fig. 2). This trend held true when the analysis was restricted only to carriers of the *CHEK2* c.1100del founder mutation. Notably, biallelic *CHEK2* PV carriers with at least one primary cancer were significantly younger at the time of diagnosis compared to monoallelic carriers (44 and 47 years, respectively; *p* = 0.0268). Similarly, homozygous carriers of *CHEK2* c.1100del with at least two cancers were significantly younger than monoallelic carriers in this category (47 and 36 years, respectively; *p* = 0.0476).

### Breast cancer risk estimates

**Fig. 3:**
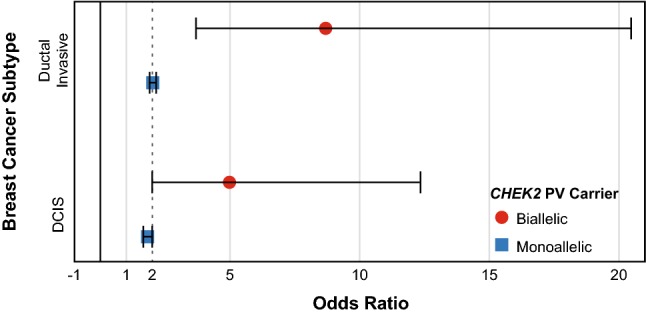
OR of developing breast cancer according to subtype and *CHEK2* PV carrier status (biallelic or monoallelic), shown with 95% CI

Multivariate logistic regression analysis was used to quantify the risk of breast cancer in biallelic *CHEK2* PV carriers and monoallelic carriers. As there were no cases of lobular invasive breast cancer among the 31 biallelic *CHEK2* PV carriers, it was not possible to determine an overall odds ratio (OR) for developing any type of breast cancer (lobular invasive, ductal invasive, and DCIS). Therefore, ORs were determined separately for the breast cancer types for which there were sufficient numbers in both biallelic and monoallelic *CHEK2* PV carriers: ductal invasive and DCIS (Fig. 3). Biallelic *CHEK2* PV carriers had an OR of 8.69 (95% CI 3.69–20.47) for ductal invasive breast cancer compared to 2.02 for monoallelic *CHEK2* PV carriers (95% CI 1.90–2.15). Similarly, biallelic *CHEK2* PV carriers had a higher risk of developing DCIS than did monoallelic carriers, with biallelic carriers having an OR of 4.98 (95% CI 2.00–12.35) and monoallelic carriers having an OR of 1.82 (95% CI 1.66–2.00). While the CI for biallelic *CHEK2* PV carriers are wide for both ductal invasive breast cancer and DCIS, the lower end of the CI range for both show at least a twofold higher risk of cancer compared to non-carriers.

## Discussion

This study compared the cancer phenotypes of women with biallelic PVs in *CHEK2* to monoallelic carriers identified by hereditary cancer panel testing. Using a similar cohort of patients who underwent clinical hereditary cancer testing, our laboratory has previously shown that women with any PV in *CHEK2* have approximately twofold greater risk of developing any breast cancer than PV-negative women [4]. This is consistent with our present findings, where the ORs for invasive breast cancer and DCIS were 2.02 and 1.82, respectively. In this cohort, biallelic *CHEK2* PV carriers had a significantly higher risk for breast cancer, were more likely to be diagnosed at or before age 50, and were more likely to have multiple primary breast cancers compared to monoallelic *CHEK2* PV carriers. Biallelic *CHEK2* PV carriers also appeared to have a higher risk of cancer overall, although we did not observe a statistically significant excess of any individual cancer other than breast. The ORs calculated for women with biallelic PVs (8.69 for invasive breast cancer and 4.98 for DCIS) suggest that biallelic *CHEK2* PV carriers could have breast cancer risks higher than those associated with PVs in *BRCA1* and *BRCA2*. It may therefore be reasonable to consider biallelic findings in *CHEK2* as high, rather than moderate, penetrance for the purposes of management.

Other breast cancer susceptibility genes have recessive phenotypes associated with biallelic PVs, including Fanconi Anemia for *BRCA2* and *PALB2,* Ataxia Telangiectasia for *ATM*, and Nijmegen Breakage Syndrome for *NBN* [13, 20–22]. *CHEK2* is distinguished by not having a defined recessive phenotype [23]. Ascertainment of cancer phenotypes relied on provider-completed TRFs, which focus on cancers for which patients meet guideline-based testing criteria. For this reason, it is possible that this study did not identify rare cancers or cancers that are not recognized for current HBOC or hereditary colorectal cancer syndrome testing criteria in North America. Therefore, this study does not rule out the possibility of other rare or common non-breast cancer phenotypes associated with biallelic inheritance of *CHEK2* PVs. Additionally, clinical information unrelated to cancers and precancerous findings were not documented on the TRF, and this study could not identify an association between biallelic *CHEK2* PVs and other phenotypes.

A possible limitation of the current study is that cancer histories were extracted from provider-completed TRFs and the reported diagnoses were not verified. Studies evaluating the accuracy of reporting of family cancer histories have shown that accuracy decreases with increasing distance in degree of relationship to the proband [24, 25]. However, self-reporting of common cancers by probands has been shown to be highly accurate [26] and the majority of the analyses presented here are based solely on the probands clinical data. Although the logistic regression model did incorporate family history information, the previously reported sensitivity analyses show that the ORs calculated using this methodology were not substantially impacted when assuming differential family history reporting [4]. Similarly, ascertainment of this sample was from a population of patients who had clinical hereditary cancer testing and this population is therefore likely to be higher risk than the general population. However, all comparisons were done within the same testing population, making the differences between groups valid regardless of baseline risk. The small sample size of the biallelic *CHEK2* PV carriers also limits this study, as certain analyses would be underpowered and could therefore not be performed. While this does not weaken the conclusions drawn from the statistically-powered analyses presented, it limits the types of analyses that could be performed.

Current breast cancer screening guidelines for women who have inherited *CHEK2* PVs include breast mammography with consideration of tomosynthesis and consideration of breast MRI starting by age 40 [12]. The findings from this study suggest that biallelic carriers of *CHEK* PVs have a considerably higher breast cancer risk than monoallelic carriers, and therefore may benefit from more aggressive management, possibly beginning at younger ages. The observed high risk for more than one primary breast cancer diagnosis in women with biallelic PVs may also have significant implications for treatment of an initial malignancy. The findings of this study add evidence to support germline testing in affected women for informing future cancer risk.
